# Human Beta Defensin 2 Selectively Inhibits HIV-1 in Highly Permissive CCR6^+^CD4^+^ T Cells

**DOI:** 10.3390/v9050111

**Published:** 2017-05-16

**Authors:** Mark K. Lafferty, Lingling Sun, Aaron Christensen-Quick, Wuyuan Lu, Alfredo Garzino-Demo

**Affiliations:** 1 Division of Basic Science, Institute of Human Virology, University of Maryland School of Medicine, Baltimore, MD 21201, USA; mark.lafferty@umaryland.edu (M.K.L.); lsun@ihv.umaryland.edu (L.S.); aac027@ucsd.edu (A.C.-Q.); wlu@ihv.umaryland.edu (W.L.); 2 Department of Microbiology and Immunology, University of Maryland School of Medicine, Baltimore, MD 21201, USA; 3 Department of Biochemistry, University of Maryland School of Medicine, Baltimore, MD 21201, USA; 4 Department of Molecular Medicine, University of Padova, Padova 35121, Italy

**Keywords:** defensins, viruses, immune response, HIV, CCR6, pathogenesis, human beta defensin 2, Th17

## Abstract

Chemokine receptor type 6 (CCR6)^+^CD4^+^ T cells are preferentially infected and depleted during HIV disease progression, but are preserved in non-progressors. CCR6 is expressed on a heterogeneous population of memory CD4^+^ T cells that are critical to mucosal immunity. Preferential infection of these cells is associated, in part, with high surface expression of CCR5, CXCR4, and α4β7. In addition, CCR6^+^CD4^+^ T cells harbor elevated levels of integrated viral DNA and high levels of proliferation markers. We have previously shown that the CCR6 ligands MIP-3α and human beta defensins inhibit HIV replication. The inhibition required CCR6 and the induction of APOBEC3G. Here, we further characterize the induction of apolipoprotein B mRNA editing enzyme (APOBEC3G) by human beta defensin 2. Human beta defensin 2 rapidly induces transcriptional induction of APOBEC3G that involves extracellular signal-regulated kinases 1/2 (ERK1/2) activation and the transcription factors NFATc2, NFATc1, and IRF4. We demonstrate that human beta defensin 2 selectively protects primary CCR6^+^CD4^+^ T cells infected with HIV-1. The selective protection of CCR6^+^CD4^+^ T cell subsets may be critical in maintaining mucosal immune function and preventing disease progression.

## 1. Introduction

HIV preferentially infects and depletes chemokine receptor type 6 (CCR6)^+^CD4^+^ T cells despite suppressive antiretroviral therapy [1,2]. The chemokine receptor CCR6 is critical for mucosal immunity [3,4,5,6,7,8,9]. CCR6 is a marker of memory CD4^+^ T cells that express the HIV co-receptors, CCR5 and CXC chemokine receptors type 4 (CXCR4), and the integrin α4β7 [2,10]. In addition, CCR6 is a marker of Th17 cells which express CXCR4 and often express CCR5 [11,12,13]. Th17 cells and the CCR6^+^CCR4^+^CD4^+^ T cell subset, which are enriched for interleukin 17 (IL-17), produce lower levels of the CCR5 ligands CCL3, CCL4, and CCL5 [2,13]. The high proportion and increased expression of CCR5 and α4β7 enhances entry and HIV envelope binding to CCR6^+^CD4^+^ T cells [2,13,14]. Further, lower expression of CCR5 ligands by CCR6^+^CD4^+^ T cells reduces self-protection and increases susceptibility [14,15]. In addition to increased susceptibility, CCR6^+^CD4^+^ T cells are more permissive to HIV-1 [16]. Independent of entry, memory CCR6^+^CD4^+^ T cells have higher levels of replication when infected with single-cycle VSVG-GFP pseudotyped HIV which was further enhanced only in CCR6^+^ cells treated with retinoic acid [17]. CCR6^+^CD4^+^ T cells, upon HIV infection are prone to apoptosis, which may contribute to their preferential depletion [1,2,13,14,17]. However, some subsets of CCR6^+^ cells may contribute to the persistent HIV reservoir [18,19,20].

The gut associated lymphoid tissue (GALT) is enriched in CCR6^+^CD4^+^ T cells including Th17 cells which are preferentially depleted in HIV-1 infection and pathogenic simian immunodeficiency virus (SIV) infection of rhesus macaques, starting during acute infection [21,22,23,24]. The severe depletion of GALT Th17 cells is not restored despite uninterrupted and suppressive highly active antiretroviral therapy which results in defects in mucosal immunity and barrier function that can lead to impaired bacterial control and bacterial dissemination [25,26,27,28,29]. In contrast, gut Th17 cells in HIV-1-infected long-term non-progressors are preserved and gut Th17 cells in non-pathogenic SIV infection are depleted but subsequently restored, and these animals do not develop AIDS or chronic immune activation [21,28,30]. These findings suggest that preservation of mucosal Th17 cells is critical to controlling microbial infections and maintaining intestinal barrier function, which may prevent microbial translocation and immune activation [21,28,31].

IL-17 producing cells are an important component of mucosal immune defenses against extracellular and intracellular bacteria and fungi, many of which are opportunistic infections observed in AIDS patients [32,33,34,35]. IL-17 plays an essential role in epithelial and mucosal defenses through the induction of pro-inflammatory cytokines, chemokines, factors involved in wound repair and enterocyte homeostasis, and is a potent inducer of human beta defensin 2 (hBD2) [36,37,38,39,40,41,42,43]. In addition, Th17 cells produce IL-22, which stimulates the production of hBD2 by epithelial cells [44,45]. Defensins are expressed at mucosal sites and exhibit broad antimicrobial activity against Gram-positive and Gram-negative bacteria, mycobacteria, fungi, enveloped viruses, and non-enveloped viruses [46,47,48,49,50,51,52,53,54,55,56,57]. In addition to direct antimicrobial properties, beta defensins recruit innate and adaptive effector cells to sites of inflammation, induce cytokines and mast cell degranulation, and aid in wound healing [58,59,60,61,62,63,64,65,66,67,68]. We and others have previously shown reduced infectivity of HIV-1 virions treated with hBDs [69,70]. We have also reported that hBD2 inhibits HIV-1 post-entry requiring the upregulation of the host restriction factor apolipoprotein B mRNA editing enzyme (APOBEC3G) [71]. APOBEC3G inhibits HIV replication involving deamination and deamination independent mechanisms and is associated with HIV control in vivo [72,73,74,75,76,77,78]. The hBD2-mediated increase in APOBEC3G depended on the expression of CCR6, the receptor used by hBD2 to induce chemotaxis of memory T cells [58,71]. We sought to identify the signaling pathway(s) that resulted in the upregulation of APOBEC3G in CCR6^+^ cells given their importance to mucosal immunity. Here, we report that hBD2 induced APOBEC3G transcripts via extracellular signal-regulated kinases 1/2 (ERK1/2) activation in concomitance with increased binding of transcription factors NFATc2, NFATc1, and IRF-4 on the APOBEC3G promoter. Further, hBD2 selectively inhibited HIV-1 replication in CCR6^+^CD4^+^ T cells, suggesting CCR6 as a target for preventive and therapeutic approaches against HIV.

## 2. Materials and Methods

### 2.1 Isolation and Culture of Primary Cells and Cell Lines

Human peripheral blood mononuclear cells (PBMCs) were isolated from leukapheresis-processed blood (NY Blood Center, New York, NY, USA) using Lymphoprep™. CD4^+^ T cells (purity of >95% as determined by flow cytometry) were isolated from unstimulated PBMCs using the Human CD4^+^ T Cell Enrichment Kit (Stem Cell Technologies Inc., Vancouver, BC, Canada). CCR6^+^CD4^+^ T cells were isolated from unstimulated CD4^+^ T cells using a CCR6-APC antibody (BD Biosciences, San Jose, CA, USA) and BD FACSAria II (BD Biosciences) performed at the Institute of Human Virology Flow cytometry core. PBMCs, CD4^+^ T cells, CCR6^+^CD4^+^ T cells, and CCR6^−^CD4^+^ T cells were maintained in complete RPMI-1640 media. Cells were activated with 10 ng/mL IL-2 and 2.5 μg/mL phytohemagglutinin (PHA) or with 5μg/mL anti-CD3 coated plates and 1 μg/mLanti-CD28 (eBioscience, San Diego, CA, USA) for 48 h and then maintained in complete RPMI-1640 media and 10 ng/mL IL-2 at a density of 1 × 10^6^ cells/mL. JKT-FT7 and JKT-FT7 CCR6 GFP cells, derivatives of the Jurkat CD4^+^ T lymphoblastoid cell line, were maintained in complete RPMI-1640 media. The JKT-FT7 and JKT-FT7 CCR6 GFP cell lines were a gift from Dr. Sam Hwang.

### 2.2. Flow Cytometry

Cells were surface stained with fluorochrome conjugated antibodies and isotype matched anti-IgG controls for 20 min. Stained cells were acquired on a FACSAria II (BD Biosciences) with a minimum of 10,000 gated events. Lymphocytes were gated based upon live/dead staining and forward and side scatter profiles. Gated populations were analyzed using FlowJo software (Tree Star, Inc., St. Ashland, OR, USA). FITC- or PE-anti-CD4, APC-or PE-anti-CCR6 were purchased from BD Biosciences.

### 2.3. Virus Production

HIV-1_BaL_ (R5 tropic) was prepared from monocyte-derived macrophages in RPMI-1640 media/human AB serum. Pseudotyped virions were generated by calcium phosphate cotransfection of 293T cells with an HIV-1 reporter virus, pNL4-3-deltaE-enhanced green fluorescent protein (EGFP) (the following reagent was obtained through the NIH AIDS Reagent Program, Division of AIDS, NIAID, NIH: pNL4-3-deltaE-EGFP (Cat# 11,100) from Drs. Haili Zhang, Yan Zhou, and Robert Siliciano) and an amphotropic murine leukemia virus (AMLV) envelope expressing plasmid.

### 2.4. Infectivity Assays

Activated PBMC, CCR6^+^CD4^+^ T cells and CCR6^−^CD4^+^ T cells (10^5^ cells/well) were infected for 2 h with 100 TCID50 of HIV-1_BaL_, or AMLV pNL4-3 ΔE-EGFP. After 2 h, cells were washed with phosphate-buffered saline (PBS, Arlington, TX, USA), and complete media was added with the appropriate treatment. Infection was monitored by p24 enzyme-linked immunoabsorbent assay (ELISA) with the use of a commercially available kit (PerkinElmer Life and Analytical Sciences, Boston, MA, USA) or by flow cytometry for GFP.

### 2.5. Immunoblotting

Cells were lysed with Radioimmunoprecipitation (RIPA) buffer (Sigma-Aldrich, Saint Louis, MO, USA) containing 1× ethylenediaminetetraacetic acid (EDTA)-free protease inhibitor cocktail (Calbiochem, Billerica, CA, USA), 0.1 mM phenylmethane sulfonyl fluoride (PMSF), and clarified by centrifugation. Total protein concentration was determined by detergent-compatible (DC) Protein Assay (Bio-Rad, Hercules, CA, USA), and equal amounts of total protein were subjected to sodium dodecyl sulfate–polyacrylamide gel electrophoresis analysis. The primary antibodies included APOBEC3G rabbit antisera (the following reagent was obtained through the NIH AIDS Reagent Program, Division of AIDS, NIAID, NIH: anti-ApoC17 from Dr. Klaus Strebel) and mouse anti–β-actin (Abcam, Cambridge, UK).

### 2.6. Quantitative RT-PCR

Activated CD4^+^ T cells, JKT-FT7, and JKT-FT7 CCR6 GFP cells (0.5 × 10^6^ cells per timepoint) were untreated or pre-treated with actinomycin D (10 μg/mL) and subsequently incubated in the presence or absence of hBD2 (20 μg/mL). RNA was extracted with the use of the RNeasy Kit with On-Column DNase digestion (Qiagen, Hilden, Germany) at the indicated timepoints. First-strand cDNA was synthesized from 500 ng total RNA with the use of the iScript cDNA Synthesis Kit (Bio-Rad). cDNA was analyzed by real-time qPCR with iQSYBR green supermix (Bio-Rad) with the following primers: APOBEC3G forward 5′-CGCAGCCTGTGTCAGAAAAG-3′ and reverse 5′-CCAACAGTGCTGAAATTCGTCATA-3′, and 18S forward 5′-ATCAACTTTCGATGGTAGTCG-3′ and reverse: 5′-TCCTTGGATGTGGTAGCCG-3′. ΔΔ*C*_t_ method was used to calculate fold change between untreated and treated cells normalized to 18S ribosomal RNA [79].

### 2.7. Fast Protein Liquid Chromatography

Cells were lysed in ice cold lysis buffer (50 mM (4-(2-hydroxyethyl)-1-piperazineethanesulfonic acid (HEPES), pH 7.4, 125 mM NaCl, 0.2% NP-40, 0.1 mM PMSF and 1× EDTA-Free protease inhibitor cocktail (Calbiochem)) and clarified by centrifugation. Total protein concentration was determined by DC Protein Assay (Bio-Rad), and equal amounts of total protein were subjected to gel filtration using a Superose 6 10/300 GL gel filtration column and AKTA Explorer (GE Healthcare, Little Chalfont, UK). Molecular weight was determined using a Gel Filtration Markers Kit for gel filtration chromatography (Sigma-Aldrich). Twenty-four 1 mL fractions were collected and equal volumes were subjected to sodium dodecyl sulfate–polyacrylamide gel electrophoresis (SDS-PAGE) analysis and probed with APOBEC3G rabbit antisera.

### 2.8. Chromatin Immunoprecipitation (ChIP) Assays

Chromatin was prepared according to the ChIP-IT Express Enzymatic protocol (Active Motif, Carlsbad, CA, USA). Immunoprecipitation was performed overnight using 2 μg of anti-NFATc1, anti-NFATc2, anti-IRF4 (Santa Cruz Biotechnology, Dallas, TX, USA), and anti-RNA Pol II and anti-IgG (Active Motif). Following incubation with antibody, DNA was eluted according to the Active Motif protocol and DNA was purified using the QIAquick PCR purification kit (Qiagen). Real-time qPCR was performed using iQSYBR green supermix (Bio-Rad) with the following primers: APOBEC3G ChIP-F 5′-GGG GAG GGG CTT GTG C-3′ and APOBEC3G ChIP-R 5′-AAG GCA ATT GCA AAG GGA A-3′. PCR was performed in triplicate with reaction conditions of 95 °C for 10 min followed by 40 cycles of 95 °C for 30 s, 60 °C for 1 min. Fold enrichment was calculated for each ChIP antibody used as quantity of ChIP DNA divided by amount of IgG DNA.

### 2.9. Total Chemical Synthesis of hBD2 and MIP-3α

hBD2 was chemically synthesized by solid-phase peptide synthesis with the use of a custom-modified procedure tailored from the previously published in situ neutralization protocol developed for Boc chemistry [59]. The β connectivity of three disulfide bonds (Cys1–Cys5, Cys2–Cys4, Cys3–Cys6) in highly pure synthetic hBD2 was independently verified by mass mapping of peptide fragments generated by enzymatic digestion and Edman degradation [59]. Correct folding of synthetic macrophage Inflammatory Protein-3 (MIP-3α) was demonstrated by the solution of its high-resolution x-ray crystal structure. Protein concentrations were determined by absorbance measurements at 280 nm with the use of molar extinction coefficients.

### 2.10. Statistical Analysis

All statistical analysis were performed using GraphPad Prism software version 5 (GraphPad Software, La Jolla, CA, USA). Statistical significance between paired samples (*p* values < 0.05 were considered significant) was calculated using a 2-tailed Student *t* test.

## 3. Results

### 3.1. CCR6^+^ Cells Are Protected by hBD2

**Figure 1:**
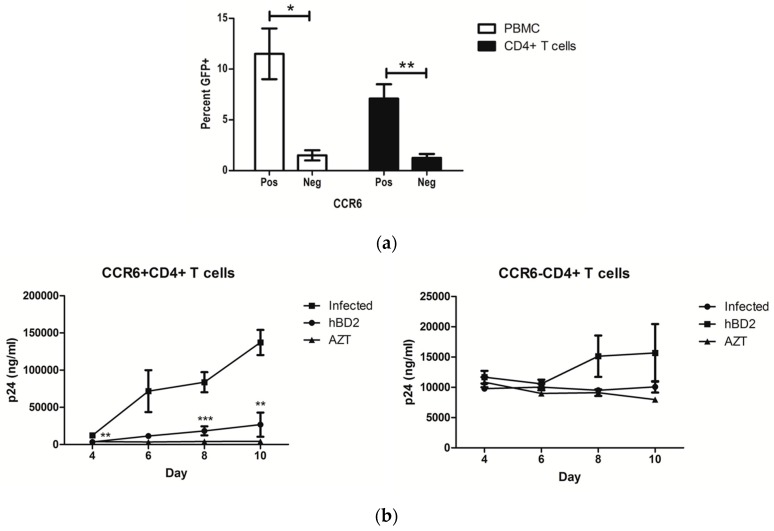
CCR6^+^CD4^+^ T cells are more permissive to HIV than CCR6^−^CD4^+^ T cells. (**a**) Peripheral blood mononuclear cells (PBMC) and CD4^+^ T cells were infected with amphotropic murine leukemia virus (AMLV) pseudotyped pNL4-3ΔE-EGFP virus for 3 days. Percent GFP^+^CD4^+^CD3^+^ cells are shown for CCR6^+^ and CCR6^−^ cells of four independent experiments (±standard error of the mean (SEM)). (**b**) CCR6^+^ and CCR6^−^CD4^+^ T cells were treated for 4 h with 20 μg/mL human beta-defensin 2 (hBD2), washed, and infected with 100 TCID50 of HIV-1_BaL_, corresponding to 1–2 ng of input p24. Some cells were treated after infection with 2.67 µg/mL of azidothymidine (AZT), for the duration of the experiment. After 6 days, HIV p24 in tissue culture supernatant was quantified by ELISA. Shown here are mean p24 ng/mL and percent inhibition (±SEM) of three independent experiments performed in triplicate. Statistical analysis was performed by paired 2-tail Student t test. * *p* < 0.05, ** *p* < 0.01, *** *p* < 0.001.

CCR6^+^CD4^+^ T cells are preferentially infected by HIV-1 and depleted [1,2]. The frequency of CD4^+^ T cells that express CCR6 varies by subtype. CCR6 is prominently expressed on peripheral blood CD45RO^+^, CCR5^+^, and IL-17 producing CD4^+^ T cells [10,11,12]. PBMC and peripheral blood CD4^+^ T cells were isolated and infected, as described in Section 2 (Materials and Methods). Consistent with reported findings, CCR6^+^ PBMC and CCR6^+^CD4^+^ T cells have higher levels of viral replication compared to CCR6^−^ cells when infected with single-cycle AMLV pseudotyped virions, which suggests the enhanced replication in CCR6^+^ cells is independent of co-receptor expression (Figure 1a). We previously reported that the CCR6 ligand hBD2 inhibits HIV-1 directly and by a post-entry mechanism during reverse transcription [70,71]. Using CCR6^+^ and CCR6^−^ Jurkat-derived cell lines, we showed that the post-entry inhibition required the expression of CCR6 [71]. To evaluate the requirement of CCR6 for inhibition in primary cells, peripheral blood CD4^+^ T cells were isolated, separated into CCR6 positive and negative fractions, and activated with anti-CD3 and anti-CD28. Cells were treated with 20 μg/mL of hBD2 for 4 h and subsequently washed three times with PBS to remove the hBD2 and infected with HIV-1_BaL_. We observed inhibition in the CCR6^+^CD4^+^ T cells but not in the CCR6^−^CD4^+^ T cells (Figure 1b).

### 3.2. hBD2 Enhances LMM and HMM APOBEC3G Expression

**Figure 2:**
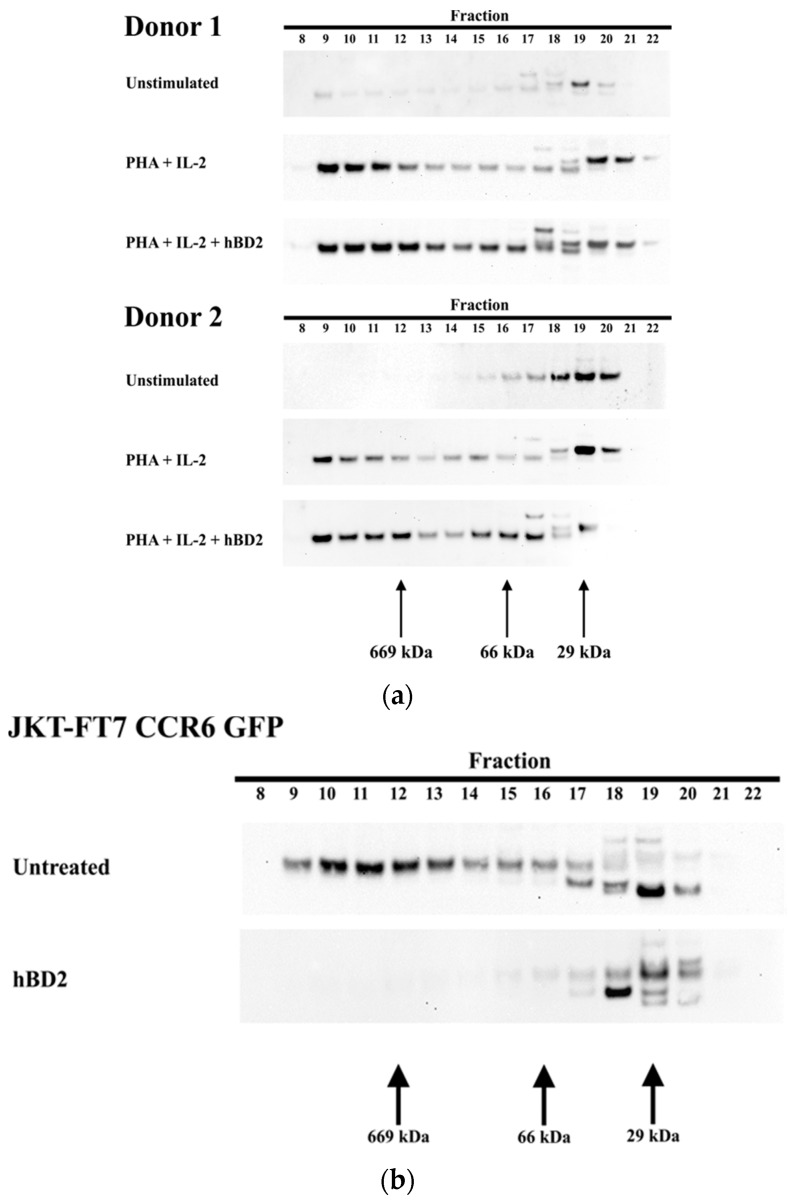
Induction of low-molecular-mass (LMM) and high-molecular-mass (HMM) APOBEC3G. (**a**) Unstimulated CD4^+^ T cells or CD4^+^ T cells activated with phytohemagglutinin (PHA) (2.5 µg/mL) and IL-2 (10 ng/mL) for 48 h and treated with hBD2 (20 µg/mL) for 8 h. (**b**) JKT-FT7 CCR6 GFP cells treated with hBD2 (20 µg/mL) for 8 h. Cell lysates were loaded on a gel size-exclusion column for fast protein liquid chromatography (FPLC) analysis. Twenty-four 1-mL fractions were collected. Eluted fractions were subjected to sodium dodecyl sulfate polyacrylamide gel electrophoresis (SDS-PAGE), followed by an immunoblotting assay with anti-apolipoprotein B mRNA editing enzyme (APOBEC3G) antibody.

Our previously published data showed that the post-entry inhibition occurred at an early stage of infection and required induction of the host restriction factor APOBEC3G [71]. We next investigated which form of APOBEC3G was present after treatment with hBD2. APOBEC3G exists in a range of molecular weight forms from a low-molecular-mass (LMM) form that restricts HIV-1 to a high molecular mass (HMM) form [80,81,82,83]. The form of APOBEC3G induced by hBD2 was determined using size exclusion chromatography. As expected, LMM APOBEC3G predominates in unstimulated CD4^+^ T cells while HMM APOBEC3G predominates in PHA stimulated CD4^+^ T cells [83,84]. Both the LMM and HMM forms of APOBEC3G exist in CD4^+^ T cells treated with hBD2 (20 μg/mL) for 8 h that were previously stimulated with PHA (Figure 2a). In JKT-FT7 CCR6 GFP cells, the predominant form was the HMM, but after treatment with hBD2 (20 μg/mL), APOBEC3G was detected only in the LMM form (Figure 2b).

### 3.3. Induction of APOBEC3G by hBD2 Requires ERK1/2 Phosphorylation

**Figure 3:**
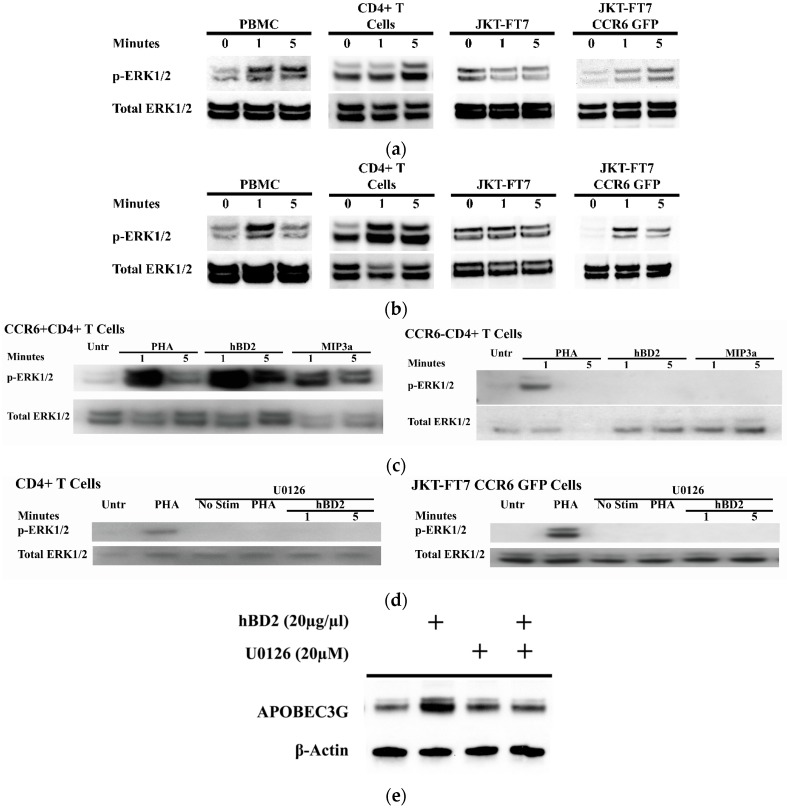
CCR6 ligands activate the extracellular signal-regulated kinases 1/2 (ERK1/2) mitogen-activated protein kinases (MAPK) pathway. Activated PBMC, CD4^+^ T cells, JKT-FT7, or JKT-FT7 CCR6 GFP cells were treated with (**a**) hBD2 (20 μg/mL) or (**b**) MIP-3α (5 μg/mL) for 1 and 5 min. Phosphorylated ERK1/2 (p-ERK1/2) and total ERK1/2 were detected at the indicated time points by Western blotting. (**c**) Activated CCR6^+^CD4^+^ T cells or CCR6^−^CD4^+^ T cells were treated with PHA (2.5 μg/mL), hBD2 (20 μg/mL) or macrophage Inflammatory Protein-3 (MIP-3α) (5 μg/mL) for 1 and 5 min. Phosphorylated ERK1/2 (p-ERK1/2) and total ERK1/2 were detected at the indicated time points by Western blotting. (**d**) Activated CD4^+^ T cells and JKT-FT7 CCR6 GFP were pre-treated with U0126 (20 µM) for 1 h and then treated with PHA (2.5 μg/mL) or hBD2 (20 µg/mL) and probed for phosphorylated ERK1/2 and total ERK1/2. (**e**) Lysates from CD4^+^ T cells pre-treated with U0126 (20 µM) and subsequently treated with hBD2 (20 µg/mL) were probed for APOBEC3G and β-actin by Western blotting. Images shown are representative of 2–4 independent experiments from different donors or cell passages.

Mitogen induction of APOBEC3G expression requires ERK1/2 activation [85,86]. To assess whether hBD2 induction of APOBEC3G involves signaling through the mitogen-activated protein kinases (MAPK) pathway, we treated activated PBMC and CD4^+^ T cells, JKT-FT7 CCR6 GFP cells, and JKT-FT7 cells, which are CCR6^−^, with hBD2 (20 μg/mL) and measured phosphorylated and total ERK1/2 by Western blot. Treatment with hBD2 increased the amount of phosphorylated ERK1/2 in PBMC, CD4^+^ T cells, and the JKT-FT7 CCR6 GFP cells (Figure 3a). Treatment with MIP-3α, the cognate ligand for CCR6, activated ERK1/2 in PBMC, CD4^+^ T cells, and JKT-FT7 CCR6 GFP cells (Figure 3b) which further supports that signaling through CCR6 activates the ERK1/2 MAPK pathway. We next determined whether signaling through CCR6 activates the ERK1/2 pathway in primary CD4^+^ T cells. Both hBD2 and MIP3α increased the phosphorylation in primary CCR6^+^CD4^+^ T cells but not in the CCR6^−^CD4^+^ T cells (Figure 3c). We then tested whether the activation of ERK1/2 by hBD2 is required for APOBEC3G induction. CD4^+^ T cells were pre-treated with U0126, a specific inhibitor of MEK1/2 activation; the only known downstream substrate of phosphorylated MEK1/2 is ERK1/2 [87]. By comparing CD4^+^ T cells and JKT-FT7 CCR6 GFP cells (Figure 3a) with U0126 pre-treated cells, we find that pre-treatment with U0126 inhibited hBD2-mediated phosphorylation of ERK1/2 in both CD4^+^ T cells and JKT-FT7 CCR6 GFP cells (Figure 3d) and induction of APOBEC3G in activated CD4^+^ T cells (Figure 3e).

### 3.4. hBD2 Induces APOBEC3G Transcription

**Figure 4:**
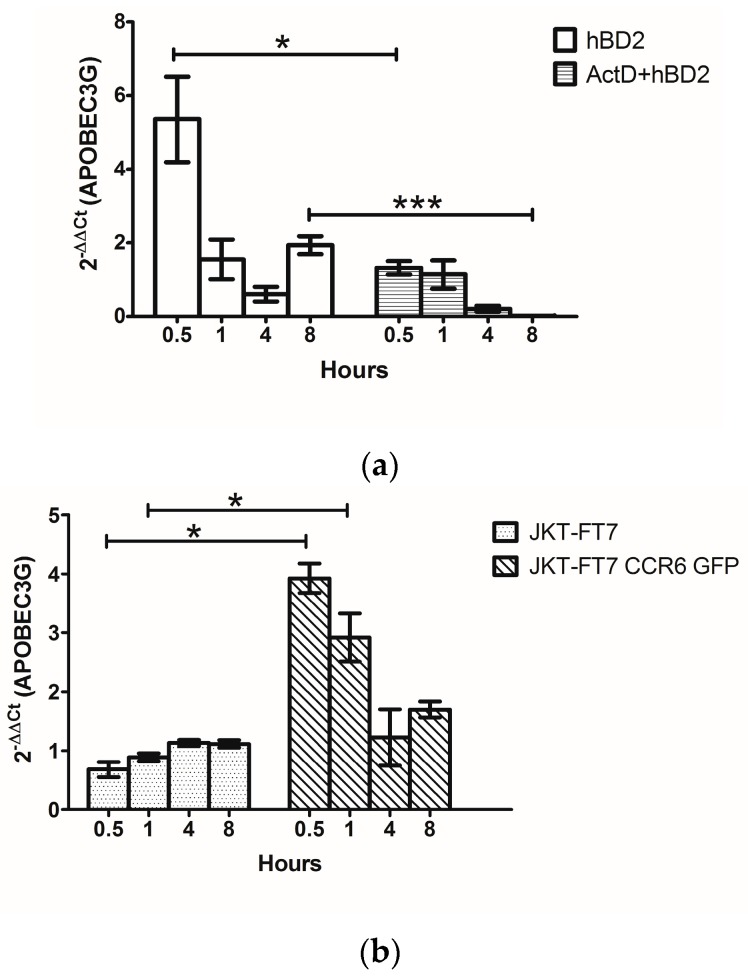
hBD2 induces transcription of APOBEC3G mRNA. (**a**) Primary CD4^+^ T cells were treated with hBD2 (20 μg/mL), pre-treated with actinomycin D (10 μg/mL), or pre-treated with actinomycin D (10 μg/mL) followed by treatment with hBD2 (20 μg/mL). APOBEC3G mRNA was assessed by quantitative real-time PCR comparing treated samples with untreated samples at matched timepoints. The data was normalized to 18S ribosomal RNA and reflects the results from two different donors. Error bars representing SEM were included. (**b**) JKT-FT7 and JKT-FT7 CCR6 GFP cells were treated with hBD2 (20 μg/mL). A3G mRNA was assessed by quantitative real-time PCR comparing treated samples with untreated samples at matched timepoints. The data reflects the results of three independent experiments. Error bars representing SEM were included. Statistical analysis was performed by paired 2-tail Student *t* test. * *p* < 0.05, *** *p* < 0.001.

We have demonstrated that hBD2 increases APOBEC3G protein expression and requires phosphorylation of ERK1/2. We previously showed that the post-entry inhibition of HIV-1 by hBD2 required the induction of APOBEC3G and CCR6 expression [71]. To determine whether the increase in APOBEC3G by hBD2 is attributable to enhanced transcription, PHA activated primary CD4^+^ T cells were treated with hBD2 (20 µg/mL). hBD2 induced both a rapid transient increase in APOBEC3G mRNA as well as a 2-fold increase at 8 h (Figure 4a). To distinguish between transcription and RNA stability, cells were pre-treated with actinomycin D (ActD). Pre-treatment with ActD abrogated the increase in APOBEC3G mRNA at all timepoints (Figure 4a). These results suggest that hBD2 induces transcription of APOBEC3G mRNA. The post-entry inhibition and induction of APOBEC3G protein by hBD2 requires CCR6, so we next tested whether the same is true for the RNA expression. JKT-FT7 cells and JKT-FT7 CCR6 GFP cells were treated with hBD2 (20 μg/mL) and APOBEC3G mRNA levels were measured by qPCR. APOBEC3G mRNA expression was compared with untreated cells at matched timepoints and normalized to 18S ribosomal RNA levels. Treatment with hBD2 increased APOBEC3G mRNA in JKT-FT7 CCR6 GFP cells but not in the JKT-FT7 cells (Figure 4b). These results are consistent with our findings that APOBEC3G protein induction is restricted to CCR6^+^ cells [71]. Similar to our findings in primary cells, we observed a rapid but transient increase in APOBEC3G mRNA following treatment with hBD2 in JKT-FT7 CCR6 GFP cells and a 2-fold increase occurring at 8 h (Figure 4b).

### 3.5. NFAT and IRF-4 Binding after hBD2 Treatment

**Figure 5:**
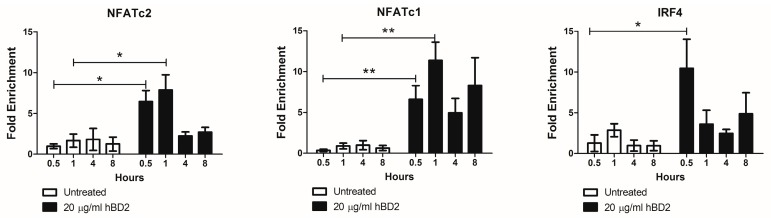
hBD2 enhances binding of NFAT and IRF-4 to the APOBEC3G promoter. JKT-FT7 CCR6 GFP cells were treated with hBD2 (20 μg/mL) for the indicated timepoints. Chromatin immunoprecipitation (ChIP) assays were performed using antibodies against NFATc2, NFATc1, or IRF-4. Immunoprecipitated DNA was detected by quantitative real-time PCR performed in triplicate. Shown is fold enrichment determined by comparison with IgG control of three independent experiments. Error bars representing SEM were included. Statistical analysis was performed by paired 2-tail Student *t* test. * *p* < 0.05, ** *p* < 0.01.

The transcription factors NFATc2, NFATc1, and IRF-4 regulate APOBEC3G in CD4^+^ T cells [88]. Therefore, we next determined by ChIP assay whether hBD2 (20 μg/mL) treatment induces NFATc2, NFATc1, and IRF-4 binding to the APOBEC3G promoter. We observed a 6.5-fold and 7.9-fold enrichment in NFATc2 and a 6.6 and 11.4-fold enrichment in NFATc1 bound to APOBEC3G promoter in hBD2 (20 μg/mL) treated JKT-FT7 CCR6 GFP cells at 30 and 60 min, respectively. hBD2 treatment also yielded a 9.5-fold enrichment of IRF-4 bound to APOBEC3G promoter at 30 min (Figure 5). Thus, the increased nuclear localization of NFAT and increased IRF-4 expression after hBD2 treatment induced transcription of APOBEC3G in JKT-FT7 CCR6 GFP cells.

## 4. Discussion

CCR6^+^CD4^+^ T cells are comprised of a heterogeneous population of cells including Th17 cells that contribute to mucosal immune defenses. CCR6 is a critical receptor in gut homeostasis that is involved in trafficking of immature DCs, B cells, and T cells to sites of inflammation and into inductive sites within the GALT. A lack of CCR6 or its ligands, MIP-3α or hBD2, is associated with immune dysfunction including reduced levels of intestinal B cells and regulatory T cells, diminished production of antigen specific IgA, and defects in T cell priming [4,5,6,7,8]. Structural defects are also associated with a lack of CCR6, MIP-3α, or hBD2 including smaller Peyer’s patches with fewer B cells and follicular domes, reduced numbers of M cells, and a block in the development of isolated lymphoid follicles [6,7,9]. Similar defects in mucosal lymphoid development are observed in the absence of MIP-3α or hBD2, suggesting non-redundant roles [3]. MIP-3α recruits immune cells to inductive sites via CCR6 and is expressed by follicle-associated epithelium and CD90^+^ Th17 cells [89,90]. Although MIP-3α inhibits HIV-1 replication in vitro, MIP-3α is strongly chemotactic for CCR6^+^ cells and therefore in vivo MIP-3α may contribute to the spread of infection by recruiting these highly susceptible cells to sites of infection [71,89]. In SIV-infected rhesus macaques, CCR6^+^CD4^+^ T cells are recruited from the periphery to the gut mucosa by MIP-3α. This disruption in CCR6^+^CD4^+^ T cell homeostasis may expand the number of highly susceptible viral targets in the gut mucosa [24]. Interestingly, glycerol monolaurate, an antimicrobial that inhibits production of proinflammatory cytokines and MIP-3α, protects rhesus macaques from mucosal SIV transmission [89]. In contrast, hBD2 may prevent the spread of infection, as it directly inhibits HIV and induces APOBEC3G, but is weakly chemotactic for CCR6^+^ cells compared with MIP-3α [70,71].

**Figure 6:**
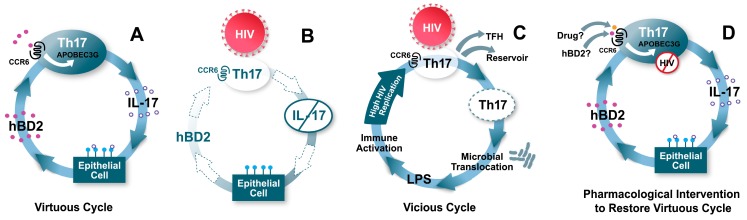
Model: hBD2 contributes to a “virtuous cycle”, maintaining mucosal integrity and protecting CCR6^+^ T cells. (**A**) CCR6^+^ T cells include Th17 cells, which produce IL-17. IL-17 has several effects on mucosal epithelia, including regulation of tight junctions, and induction of the antimicrobial peptide hBD2. hBD2 binding to CCR6 induces intracellular signaling events leading to increased transcription of APOBEC3G, which inhibits HIV intracellularly. (**B**) When HIV reaches CCR6^+^ Th17 cells due to breaches in the mucosa, their death causes loss of production of IL-17, and hBD2 production is also decreased as a consequence. Thus, CCR6^+^ T cells become more permissive to HIV infection. (**C**) The loss of mucosal integrity due to lower levels of Th17 and hBD2 result in microbial translocation. High levels of lipopolysaccharide (LPS) and other microbial factors cause immune activation, which further enhance HIV replication, eventuating in a self-perpetuating “vicious cycle”. (**D**) Induction or administration of adequate levels of hBD2, or a pharmaceutical drug targeting CCR6 could result in high levels of expression of APOBEC3G and protection of CCR6^+^ Th17 cells.

CCR6^+^CD4^+^ T cells and Th17 cells are redistributed from peripheral blood and preferentially depleted from the GALT, which correlates with disease progression [1,2,13,14,17,21,24,90]. The connections between CCR6, its ligands, and Th17 cells are multiple and highly relevant in the context of HIV infection. Th17 cells are an important component of mucosal immune defenses against extracellular and intracellular bacteria and fungi [32,34]. IL-17 limits microbes through the induction of pro-inflammatory cytokines, chemokines that recruit innate and adaptive immune cells, proteins involved in enterocyte homeostasis and antimicrobial peptides such as hBD2 [36,37,38,39,41,42,91,92]. Limiting microbial threats, maintenance, and re-establishment of the mucosal barrier are vital for intestinal homeostasis and prevention of microbial translocation. It has been shown that CCR6^+^CCR4^+^, CCR6^+^CXCR3^+^ and CCR6^+^CD90^+^ Th17 cell subsets are highly permissive to HIV-1 infection [2,90]. Th17 cells can also convert into follicular T helper cells (T_FH_), a cell type that is highly infectible by HIV and SIV, despite expressing low levels of CCR5 [93,94,95,96]. It is possible that Th17 CCR6^+^ cells may get initially infected, and then convert to T_FH_ cells. Interestingly, CCR6^+^CCR4^+^ Th17 cells are specific for *Candida albicans* and CCR6^+^CXCR3^+^ Th17 cells are specific for *Mycobacterium tuberculosis*, two HIV-1 associated infections [11]. Thus, the preferential depletion of CCR6^+^ T cells may be a key event in the pathogenesis of HIV infection (Figure 6) [11,97].

We have previously reported that hBD2 inhibits HIV-1 replication post-entry requiring induction of APOBEC3G via CCR6 [71]. Here, we found that hBD2 selectively inhibits HIV replication in CCR6^+^ CD4^+^ cells, but not in CCR6^−^CD4^+^ cells. As expected, the levels of HIV replication in CCR6^−^CD4^+^ cells were significantly lower than in CCR6^+^CD4^+^ cells; however, that low level of replication could not be inhibited by treating cells with azidothymidine (AZT), a reverse transcriptase inhibitor. One intriguing possibility is that this low level of reverse-transcription independent replication may lead to HIV latency, making the CCR6^−^CD4^+^ cells a possible reservoir. In this study, we analyzed the status (LMM versus HMM) of APOBEC3G and identified the hBD2-induced signaling pathway and transcription factors that result in its increased expression and selective protection of CCR6^+^CD4^+^ cells. We found that hBD2 causes a dramatic shift from the HMM to the highly active LMM form in CCR6^+^ Jurkat cells, but not in primary CD4^+^ T cells. It is possible that the lack of shift from HMM to LMM is due to the lack of depolimerization in CCR6^−^ cells, however due to cell number limitations, the molecular mass of APOBEC3G was not determined in primary cells separated into CCR6^+^ and CCR6^−^ subsets. Alternatively, it is possible that differences in signaling pathways between Jurkat and primary cells may be responsible for this difference. Treatment with hBD2 resulted in ERK1/2 phosphorylation in PBMC, CD4^+^ T cells, and JKT-FT7 CCR6 GFP cells and was required for APOBEC3G expression. We observed rapid transcriptional activation of APOBEC3G, which was sensitive to treatment with the transcriptional inhibitor actinomycin D, in both primary CD4^+^ T cells and JKT-FT7 CCR6 GFP cells. In contrast, hBD2 did not induce APOBEC3G in the CCR6^−^ JKT-FT7 cells. The rapid transcriptional induction of APOBEC3G mRNA confirms the results from our ChIP experiments. We found enrichment of APOBEC3G promoter sequence pulled down with NFATc2, NFATc1, and IRF-4 antibodies at early timepoints. NFAT proteins cooperate with additional transcription factors and Farrow et al. identified an NFAT/IRF-4 composite site in the APOBEC3G promoter [88]. NFAT and IRF-4 induce APOBEC3G expression, which is markedly enhanced upon cooperative binding [88]. Our results suggest that the early increase in APOBEC3G mRNA after hBD2 treatment is due to the presence of both NFAT and IRF-4.

The preferential infection of CCR6^+^ Th17 cells may be attributable to the higher expression of the viral receptors CD4, CXCR4, and α4β7 compared with CCR6^−^ Th17 cells resulting in enhanced HIV envelope binding [14]. Despite similar CCR5 expression levels, CCR6^+^ Th17 cells may be more susceptible to R5 tropic virus, partly due to low expression of CCR5 ligands and decreased expression of antiviral factors [14,16]. The preferential infection and depletion of CCR6^+^CD4^+^ T cell subsets may initiate or contribute to the failure of the gut mucosal immune system. HIV-1-infected long-term non-progressors and SIV-infected sooty mangabeys, which do not progress to AIDS and lack both microbial translocation and chronic immune activation, maintain levels of Th17 cells in both the blood and gut [21,28,30]. These findings suggest that protecting CCR6^+^CD4^+^ T cell subsets may be critical in preventing disease progression, and hBD2 produced from mucosal epithelial cells could contribute to preserving this subset (Figure 6). Consistent with this hypothesis, a recent study has highlighted that hBD2 is a key factor differentially expressed in primary endometrial epithelial cells (which produce protective factors), as compared to endometrial stromal fibroblasts (which enhance HIV infection in vitro) [98].

In agreement with previously reported findings, we found that CCR6^+^CD4^+^ T cells have higher levels of replication when infected with pNL4-3-deltaE-EGFP HIV pseudotyped with the AMLV envelope which does not use HIV receptors or CCR6 for entry [16,17]. In addition to the previously reported elevated levels of HIV receptors and decreased CCR5 ligands, these findings suggest that additional post-entry cellular factors contribute to the preferential infection of CCR6^+^ cells. The CCR6-mediated intracellular inhibition of HIV-1 by hBD2 described herein, provides insight into signaling pathways that may guide the development of therapeutics that selectively target and protect CCR6^+^ cells.

## 5. Conclusions

Our study shows that hBD2 protects CCR6^+^CD4^+^ T cells from HIV infection. The protection is associated with increased APOBEC3G transcription, due to ERK1/2 signaling. Since there is increasing evidence that CCR6^+^CD4^+^ cells are lost early in HIV infection, our results are important to devise novel strategy to combat HIV infection. One attractive possibility is to design drugs that bind to CCR6 inducing APOBEC3G transcription. hBD2 has low chemotactic activity, so that it is conceivable that a novel CCR6-binding drug could induce APOBEC3G expression, and have low chemotactic index. Such drug could target HIV even in the latent reservoir, which has been shown to include CCR6^+^ cells.
